# Prevalence of Cognitive Defects Among Senior Inmates of Old-Age Homes in a District of South Kerala

**DOI:** 10.7759/cureus.65082

**Published:** 2024-07-22

**Authors:** Anish J Philip, Felix Johns

**Affiliations:** 1 Department of Community Medicine, PK Das Institute of Medical Sciences, Kerala, IND; 2 Department of Community Medicine, Pushpagiri Institute of Medical Sciences and Research Centre, Kerala, IND

**Keywords:** kerala, elderly population, old-age homes, sociodemographic factors, prevalence, cognitive impairment

## Abstract

Background

Cognitive decline, including mild cognitive impairment and major neurocognitive disorder, is a growing concern among aging populations. The prevalence of these disorders is especially relevant in regions experiencing a surge in elderly care infrastructure, such as Kerala, India. Within the context of sociocultural shifts and a rising elderly population, old-age homes are increasingly becoming the focal point of care and support for cognitive health. This study aims to examine the prevalence of cognitive defects among elderly individuals residing in old-age homes in the Kottayam district of Kerala, India. Additionally, it investigates the relationship between cognitive disorders and sociodemographic variables of the inhabitants in these institutionalized settings.

Methodology

In this study, 535 elderly subjects were selected from 85 registered old-age homes in Kottayam, Kerala, through systematic cluster sampling. The response rate was 89.6%. Data collection involved a sociodemographic questionnaire, the Picture Memory Impairment Screen (PMIS), and the Mini-Mental Status Examination (MMSE) translated into Malayalam. Data were gathered either from participants, caretakers, or medical records. Ethical guidelines were strictly followed. Statistical analysis was conducted using SPSS Statistics Version 25.0 (IBM Corp., Armonk, NY, USA), employing the chi-square test, t-test, and logistic regression, with a p-value less than 0.05 deemed significant.

Results

In our study of 535 elderly individuals in Kottayam’s old-age homes, age, gender, and education were significantly associated with cognitive impairment, with p-values <0.001, 0.049, and <0.001, respectively. Behavioral factors such as smoking and alcohol consumption showed no significant association. The mean MMSE and PMIS scores were 25.24 and 5.57, respectively. The prevalence of cognitive defects was 170 (31.80%) as per MMSE and 182 (34.00%) according to PMIS. Given the wider acceptance of MMSE, the study established a cognitive defect prevalence of 170 (31.80%) among senior inmates.

Conclusions

This study reveals a high prevalence (170, 31.80%) of cognitive defects among elderly residents in Kottayam’s old-age homes. Age and education were significant predictors, while behavioral factors such as smoking and alcohol were not. These findings underscore the need for targeted healthcare strategies to address cognitive decline in aging populations.

## Introduction

Cognition, encompassing intellect, memory, executive function, language, and decision-making, is a fundamental aspect of human function [1]. Even in states of rest, our brains actively process various stimuli. However, as humans age, cognitive abilities might deteriorate, leading to conditions termed dementia or, in milder cases, mild cognitive impairment (MCI). Today’s terminology leans toward terms such as major or minor cognitive disorder instead of dementia [2]. MCI is an intermediate stage, and its symptoms can range from increased forgetfulness to trouble navigating familiar surroundings [3]. Major neurocognitive disorder, previously called dementia, includes impairments in various cognitive domains such as memory, language, and self-control. Multiple conditions, including Alzheimer’s disease and vascular cognitive impairment, can lead to such disorders. Diagnosis primarily depends on clinical judgment due to a lack of definitive biomarkers in living patients [4]. Aging remains the primary risk factor for cognitive issues. Although a degree of cognitive decline is normal with age, significant deterioration impacts an individual’s ability to manage daily life. The natural physical and functional transformations of the brain that come with age can lead to challenges in memory and problem-solving [5].

The global rise in the aging population brings attention to the care and well-being of the elderly. Institutions such as old-age homes are becoming more prevalent, particularly in countries undergoing sociocultural changes, such as India. Kerala, a state in India, has seen a considerable rise in the number of elderly individuals seeking refuge in old-age homes. This change is emblematic of a broader global trend, where the growing elderly population in developing nations grapples with cognitive disorders and the infrastructure required to support them [6]. Our study focused on examining the prevalence of cognitive defects among elderly individuals residing in institutionalized centers, specifically old-age homes, in the district of Kottayam in Kerala. Our study also determined the association between cognitive defects and the sociodemographic variables of inhabitants in old-age homes in a district of south Kerala.

## Materials and methods

This study utilized a systematic cluster sampling technique across the 85 registered old-age homes in the Kottayam district, accounting for a total of 3,013 senior inmates. Each cluster was designated a size of five, resulting in 107 clusters and a sample size of 535 subjects. There was an impressive response rate of 89.6%. To ensure the validity of the gathered data, eight old-age homes were randomly selected for a re-evaluation. Ethical committee approval was obtained from the Institutional Ethical Committee of Pushpagiri Institute of Medical Sciences and Research Centre (PIMSRC/E1/388A/36/2017).

Study tools

A sociodemographic questionnaire covered various domains such as age, gender, living family members, education status (as per the Modified Kuppuswamy’s Scale), previous occupation, monthly income, marital status, past harmful habits, and any family history of dementia. The Picture Memory Impairment Screen (PMIS) involved presenting participants with photos of four distinct objects. They were later prompted to recall these objects, either freely, with a cue, or not at all, and scores were assigned based on their recall ability. Finally, a Mini-Mental Status Examination (MMSE) questionnaire was used. It was a detailed 11-question assessment that was tailored for the local demographic by being translated into Malayalam. Data collection strictly followed ethical guidelines. After obtaining the necessary approvals and informed consent from participants, interviews were conducted using a pre-tested, semi-structured questionnaire. In scenarios where subjects could not respond, data were extracted either from their caretakers or from available medical records. The cognitive abilities of the participants were assessed using both the PMIS and the MMSE. On average, collecting all relevant data from a subject took approximately 20 minutes.

The main variable of interest in the study was cognitive ability, gauged using the MMSE score. Numerous co-variates were also considered, such as age, gender, education, occupation, monthly income, marital status, and various health metrics, including the presence or absence of specific ailments. For clarity in data interpretation, the study also defined operational terms; a “senior inmate” was an individual aged 60 or above residing in a registered old-age home, and “cognitive impairment” was determined based on the scores from the PMIS and MMSE. Ethical considerations were paramount. Necessary clearances were acquired, and the confidentiality of all participants was preserved throughout the study. Regarding budget, the primary expenses were associated with travel (INR 42,500) and resources such as books and printing (INR 10,000). The researchers took the responsibility for all costs.

Statistical analysis

For data analysis, all the gathered data were coded and transferred to Microsoft Excel (Microsoft Corp., Redmond, WA, USA) and subsequently analyzed using SPSS Statistics Version 25.0 (IBM Corp., Armonk, NY). Various statistical methods, such as the chi-square test, t-test, and logistic regression, were employed. A p-value less than 0.05 was the benchmark for statistical significance.

## Results

**Figure 1:**
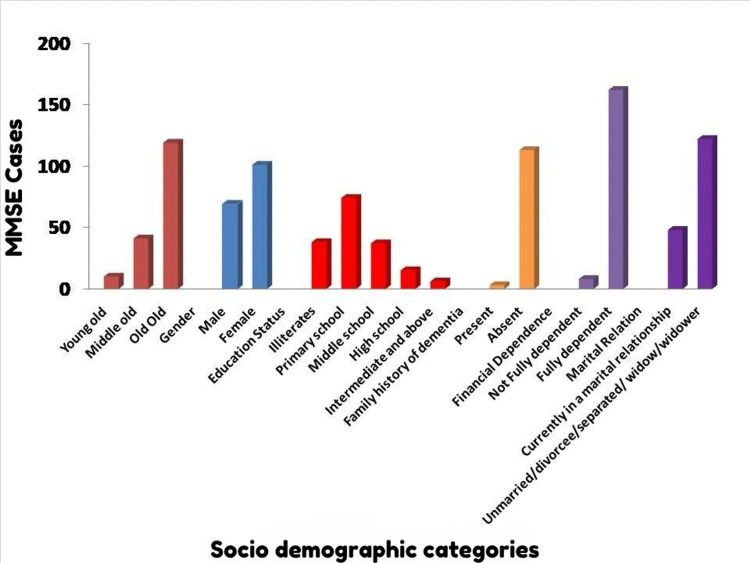
Univariate analysis of sociodemographic characteristics with cognitive impairment among the study population (n = 535). MMSE = Mini-Mental Status Examination

Based on Table 1 and Figure 1, which present a univariate analysis of sociodemographic characteristics of cognitive impairment among a study population of 535 individuals, the following results can be inferred: age appeared to be significantly associated with cognitive impairment. In the “Young old” category, 184 individuals were found to be without cognitive defects, while only 10 had cognitive impairment. The “Middle old” category had 118 individuals without cognitive defects and 41 with defects. The “Old Old” category demonstrated a more pronounced presence of cognitive defects, with 119 out of 182 individuals showing impairment. The chi-square value for age was found to be 160.88, with a highly significant p-value <0.001. Regarding gender, 162 males did not have cognitive defects while 69 did. Among females, 203 were without cognitive defects compared to 101 with defects. The chi-square value for gender was 0.681, which was statistically significant, with a p-value of 0.049. Education status also showed a significant association with cognitive impairment. The chi-square value for this category was 39.86, with a significant p-value <0.001. Illiterates had a higher proportion of cognitive defects (38 out of 67), followed by those who attended primary school, with 74 out of 199 individuals showing cognitive defects. As the education level increased, the number of individuals with cognitive defects decreased. A family history of dementia did not seem to have a significant association with cognitive impairment. Five individuals with a family history of dementia did not have cognitive defects while three did. Meanwhile, 243 individuals without a family history did not exhibit cognitive defects while 113 did. The chi-square value for this category was 0.119 with a non-significant p-value of 0.730. Financial dependence also did not show a significant relationship with cognitive impairment. For those not fully dependent, 15 were without cognitive defects compared to eight with defects. Those fully dependent had 350 individuals without defects and 162 with defects, yielding a chi-square value of 0.100 and a p-value of 0.752. Lastly, marital status did not seem to be significantly associated with cognitive impairment. Overall, 184 individuals currently in a marital relationship did not have cognitive defects while 48 did. For those unmarried, divorced, separated, or widowed, 181 were without defects, and 122 exhibited cognitive defects. The chi-square value for marital relations was 0.623, with a p-value of 0.430.

**Table 1:** Univariate analysis of sociodemographic characteristics with cognitive impairment among the study population (n = 535). A p-value less than 0.05 was the benchmark for statistical significance.

Category	Cognitive defect absent	Cognitive defect present	Chi-square value	P-value
Age				
Young old	184	10	160.88	<0.001
Middle old	118	41		
Old old	63	119		
Gender				
Male	162	69	0.681	0.049
Female	203	101		
Education status				
Illiterates	29	38	39.86	<0.001
Primary school	125	74		
Middle school	95	37		
High school	82	15		
Intermediate and above	34	6		
Family history of dementia				
Present	5	3	0.119	0.730
Absent	243	113		
Financial dependence				
Not fully dependent	15	8	0.100	0.752
Fully dependent	350	162		
Marital relationship				
Currently in a marital relationship	184	48	0.623	0.430
Unmarried/Divorced/Separated/Widowed/Widower	181	122		

**Figure 2:**
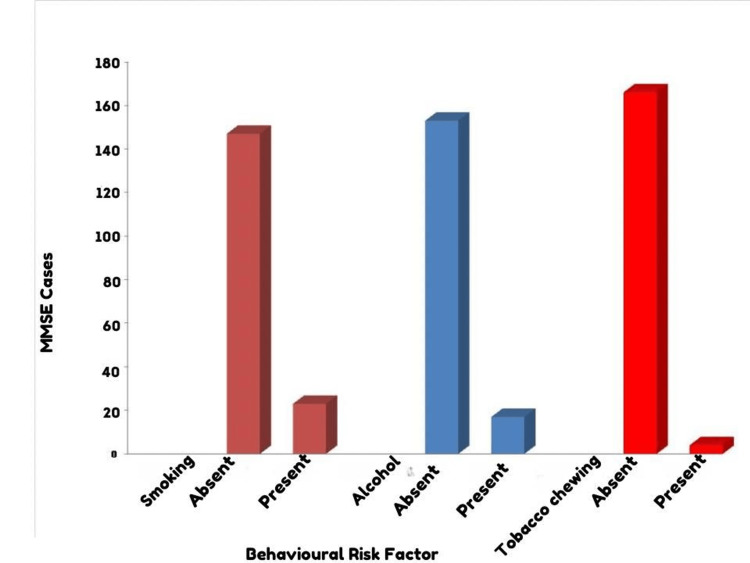
Association between behavioral risk factors with cognitive impairment. MMSE = Mini-Mental Status Examination

Based on Table 2 and Figure 2, which assesses the association between behavioral risk factors and cognitive impairment, for smoking, among those who did not smoke, 324 individuals did not have cognitive impairment while 147 did. Among the smokers, 41 were without cognitive defects, and 23 had them. The chi-square value for the association between smoking and cognitive impairment was 0.581, and the p-value was 0.446, indicating no significant association between smoking and cognitive defects. Regarding alcohol consumption, 336 non-alcohol consumers did not show signs of cognitive impairment while 153 did. Among those who consumed alcohol, 29 were free from cognitive defects, and 17 had them. The chi-square value for this association was 0.623, with a p-value of 0.430, suggesting that alcohol consumption was not significantly associated with cognitive impairment. For the behavior of tobacco chewing, the majority, i.e., 362 non-tobacco chewers, did not have cognitive defects, whereas 166 did. In contrast, among those who chewed tobacco, three were without cognitive impairment, and four exhibited signs of it. The chi-square value for tobacco chewing’s association with cognitive impairment was 2.105, with a p-value of 0.147. This indicates that there was no significant relationship between tobacco chewing and cognitive defects in the studied population.

**Table 2:** Association between behavioral risk factors with cognitive impairment. A p-value less than 0.05 was the benchmark for statistical significance.

Behavioral risk factors	Cognitive impairment		Chi-square value	P-value
	Absent	Present		
Smoking absent	324	147	0.446	0.446
Smoking present	41	23		
Alcohol absent	336	153	0.430	0.430
Alcohol present	29	17		
Tobacco chewing absent	362	166	2.105	0.147
Tobacco chewing present	3	4		

The mean value of the PMIS score for 535 inmates was 5.57 with a standard deviation of 2.155, and the MMSE score for 535 inmates was 25.24 with a standard deviation of 4.153. The 50th percentile value of the PMIS score was 6 and the MMSE score was 27 (Table 3).

**Table 3:** Details of the Picture Memory Impairment Screen score and Mini-Mental Status Examination score.

Parameters		Mini-Mental Status Examination score	Picture Memory Impairment Screen score
Mean		25.24	5.57
Standard deviation		4.153	2.155
Percentiles	Q1	22.00	4.00
	50	27.00	6.00
	Q3	28.00	7.00

**Figure 3:**
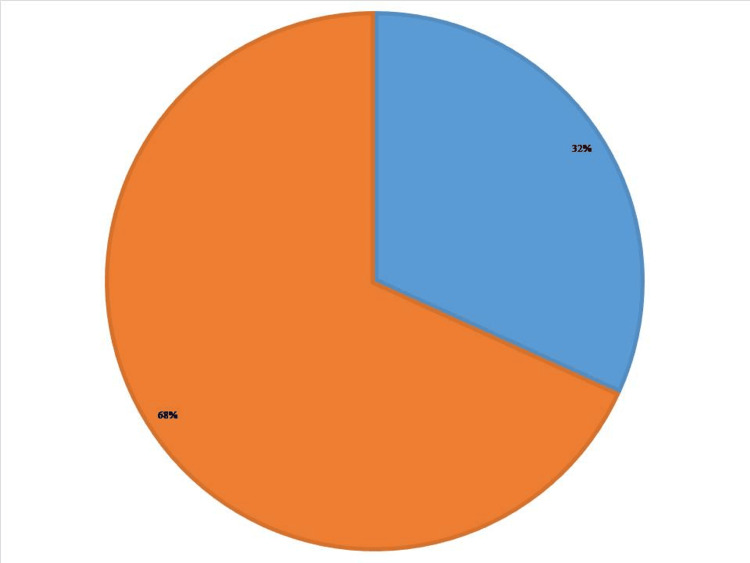
Pie chart of the prevalence of cognitive defects as per the MMSE score in old-age homes of Kottayam district. Blue: 32%, cognitive defect present as per MMSE. Orange: 68%, cognitive defect absent as per MMSE. MMSE = Mini-Mental Status Examination

A total of 182 (34%) study subjects had cognitive defects among 535 on the PMIS test while 170 (31.80%) had cognitive defects on the MMSE test. As MMSE is a more valid and widely accepted neuropsychological tool, we fixed the prevalence of cognitive defects among senior inmates of old-age homes in Kottayam district at 170 (31.80%) (Table 4, Figure 3).

**Table 4:** Prevalence of cognitive defects.

Test method	Number of subjects with cognitive defects	Total study subjects	Prevalence (%)
Picture Memory Impairment Screen	182	535	34
Mini-Mental Status Examination	170	535	31.80

## Discussion

This study aimed to evaluate the prevalence of cognitive defects among senior inmates of old-age homes in a district of south Kerala and explore the association between cognitive defects and sociodemographic variables and behavioral risk factors.

Sociodemographic characteristics and cognitive impairment

Age and Cognitive Impairment

In this study, participants were predominantly seniors with a mean age of 74.56 years. The study cohort was classified into the following three age sub-groups, as established by previous studies: the young old (60-69 years), accounting for 194 (36.3%) individuals; the middle old (70-79 years), accounting for 159 (29.7%) individuals; and the very old (80+ years), accounting for 182 (34%) individuals [7,8]. Remarkably, the age range extended up to 98 years, highlighting the caregiver burden and the associated health challenges in old-age homes. This finding is particularly significant in the context of Kerala, which boasts the highest average life expectancy (74.9 years) among Indian states as of 2014 SRS studies [9].

One interesting observation was that some elderly participants related their age to significant historical events, such as India’s independence, rather than knowing their exact age. This lack of age awareness could be partly attributed to educational gaps or cultural factors. Age is a pivotal, non-modifiable risk factor for cognitive impairment or dementia, a conclusion widely supported in scientific literature. Cognitive decline is seen to be more prevalent with aging in both males and females. Factors such as reduced brain volume, cortical thinning, loss of myelin integrity, and diminished secretion of neurotransmitters such as acetylcholine have been implicated in this cognitive deterioration [10].

Statistical analysis in our study demonstrated that for each additional year in age, there was 1.189 times the odds of developing cognitive defects, indicated by a p-value of less than 0.001 (95% confidence interval (CI) = 1.155-1.255). These findings align with another Indian study using the MMSE scale, which reported an adjusted odds ratio of 2.98 (95% CI = 1.20-7.38) for individuals aged 60 or above, with a p-value of 0.018 [11]. Multiple forward logistic regression also confirmed age as a significant predictor with an odds ratio of 1.19 (95% CI = 1.136-1.246, p < 0.001). Therefore, age remains a critical factor for developing cognitive impairment, underscoring the need for age-specific healthcare strategies.

Gender and Cognitive Impairment

The study encompassed 535 subjects, of whom 231 (43.2%) were male and 304 (56.8%) were female. This higher female prevalence in Kottayam’s old-age homes can be attributed to multiple factors, including greater life expectancy in women (77.8 years) as opposed to men (72 years), according to 2014 SRS data [9]. Additionally, the sex ratio in Kerala is 1,084 females per 1,000 males, compared to the national average of 940 [10]. Many females faced socioeconomic challenges after widowhood, often exacerbated by a lack of employment and familial support. Some old-age homes in the district are female-exclusive, further increasing female representation. Our data hinted at a marginal gender difference in cognitive impairment vulnerability, supported by a p-value of 0.049, aligning with global studies that also note slight gender disparities due to hormonal, genetic, and cultural variables.

Education and Cognitive Impairment

In this study, we found that 67 (12.5%) were illiterate, far higher than one would expect given Kottayam’s impressive literacy rate of 97.48%, according to the 2011 Census [9,12]. This discrepancy also stands out considering Kerala’s top position in the Educational Development Index among Indian states in 2006-2007 [13]. Interestingly, only 97 (25.7%) had a high school education or more, compared to a 2001 report stating that 45% of Kerala’s elderly had at least a high school certificate [14].

Education emerged as a pivotal predictor for cognitive health, aligning with the cognitive reserve theory, which proposes that a stimulating intellectual environment can delay the onset of cognitive symptoms, even in the presence of underlying brain pathology [15]. In our data, educational status had a significant inverse relationship with cognitive impairment (p < 0.001). Using logistic regression analysis and setting middle school education as the reference level, we found that being illiterate dramatically increased the risk of cognitive defects with an odds ratio of 3.364 (95% CI = 1.820-6.221, p < 0.001).

It is essential to note a potential bias in our findings, as we maintained the MMSE score cutoff at 23 for all educational levels, which might disproportionately affect the lower-educated segment in cognitive assessments. Nevertheless, our results echo existing literature suggesting that education holds a negative and independent correlation with cognitive decline [16,17]. Kerala’s social development, particularly in education and literacy, has historical roots in Christian missionary activities that emphasize Western education. This long-term focus on education in the state impacts individual life choices and values, further emphasizing the significance of education as a determinant of cognitive health in this aging population.

Family History of Dementia and Cognitive Impairment

In our cohort, a mere nine (1.5%) reported a family history of dementia, while a significant 171 (32%) were uncertain about their family’s medical history. This lack of awareness is likely due to long-standing estrangement from their biological families. Compared to broader data, which indicates a 3% prevalence rate of dementia or significant cognitive impairment among those above 55 in Kerala [18], the incidence in our study sample appears to be lower. This gap may partly be due to the high percentage of participants who have lost contact with their families, thus leading to uncertainty about hereditary factors related to cognitive health.

Financial Dependence and Cognitive Impairment

In Kottayam district, a significant 512 (95.70%) lacked personal income and relied entirely on old age homes for financial support. In contrast, a mere 23 (4.30%) had a monthly income between INR 8,000 and INR 30,000. The vast majority of the 85 old-age homes in the district, except for two, provided shelter to the elderly on a charitable basis, charging no fees. Luxury retirement homes, which are emerging as a popular trend in various parts of India, including the Cochin region of Kerala, have not yet made a significant impact in Kottayam district. These luxurious facilities are not typically categorized as traditional old-age homes.

The less affluent individuals, primarily residing in Kottayam’s registered old-age homes, often have no ties with their families. This disconnection places them at heightened risk for potential mistreatment. A notable observation is the lack of awareness among many inmates about old-age pension programs like the Indira Gandhi National Old Age Pension. The majority of these homes operate primarily through charitable contributions from associated non-governmental organizations, combined with assistance from the Social Justice department for essentials such as food supplies.

Marital Status and Cognitive Impairment

In the participant pool, 233 (43.4%) were currently married, while the remaining 302 (56.6%) were either single, divorced, separated, widowed, or otherwise not in a marital relationship. Notably, a considerable number among those classified as married had lost contact with their spouses but had not formally separated. Additionally, some participants concealed their true marital status, potentially due to social stigmas associated with being divorced or separated. This suggests that the reported marital statuses may not fully reflect the complexity of the participants’ actual relationship statuses.

Behavioral Risk Factors and Cognitive Status

The study examined substance use among elderly residents, finding a low prevalence of tobacco and alcohol consumption, contrasting sharply with previous research. According to a previous report, nearly 25% of Kerala’s elderly population had tobacco-chewing habits, and similar rates were reported for smoking and alcohol use [14]. In our study, only seven (1.3%) had previously chewed tobacco, 64 (12%) had smoked, and 46 (8.6%) had consumed alcohol. The significantly lower rates might be attributed to the high proportion of female participants, who generally have fewer such habits compared to the more balanced gender distribution in the broader population.

Smoking and Cognitive Impairment

The non-significant association between smoking and cognitive defects in our study is intriguing. Some global studies suggest smoking as a risk factor for dementia, yet in our population, the association was not marked, possibly due to underreporting or other overriding regional factors.

Alcohol Consumption and Cognitive Impairment

Similarly, while some studies have demonstrated both protective and harmful effects of alcohol on cognitive health, our study did not find a significant association. Cultural norms and drinking patterns might influence this result [14].

Tobacco Chewing and Cognitive Impairment

Our findings also did not indicate a significant association between tobacco chewing and cognitive defects. While globally, tobacco use is cited as a risk factor for dementia, our results suggest that in the context of this district in Kerala, other factors might play a more dominant role.

Prevalence of Cognitive Defects

Our investigation used PMIS and MMSE assessments to gauge cognitive health among 535 seniors living in Kottayam’s old-age homes. A prevalence rate of 170 (31.8%) for cognitive impairment was found using MMSE, aligning with another study from Kerala which reported a combined prevalence rate of 35.92% for mild and major impairments [18]. Compared to data from Kolkata, our rates are higher but consistent with those from Chennai and Mangaluru [19-21]. We identified age and education as substantial determinants of cognitive health, affirming findings from global research. While the influence of age was anticipated, the role of education in cognitive health was particularly notable. Gender differences were observed, albeit subtly, and require additional studies.

Limitations

The study’s limitations encompass its cross-sectional design, precluding causal inferences between sociodemographic factors and cognitive impairment. The employment of the MMSE and PMIS, despite their widespread use, may not fully encompass the complexities of cognitive function. Furthermore, the study’s focus on elderly residents of old-age homes in Kottayam limits the generalizability of findings to broader elderly populations in diverse geographic regions or living arrangements. Additionally, reliance on self-reported data for variables such as smoking and alcohol consumption introduces potential biases and inaccuracies. This study provides valuable insights into cognitive impairment among the elderly in Kottayam’s old-age homes but is constrained by limitations that affect the interpretation of its findings and their broader applicability. The use of the MMSE and PMIS while translated into Malayalam, may not fully grasp the cognitive nuances within these culturally and educationally diverse groups. Regional dialects and cultural expressions could play a role in the translations and lead to inaccuracies in assessing cognitive states. The reliance on self-reported data for lifestyle factors such as smoking and alcohol consumption, where memory impairment among participants could affect the accuracy. Extrapolating these findings to broader populations may not reflect those in other regions as they could differ significantly in terms of healthcare access and social support systems. These factors underscore the need for a cautious interpretation of the results and emphasize the importance of replicating this research in varied settings to confirm these findings and assess their relevance across different demographic and regional contexts.

## Conclusions

This comprehensive study sheds critical insights on the prevalence and factors influencing cognitive impairment among elderly residents of old-age homes in Kottayam district, Kerala. Our findings underline the significant roles of age and educational level as primary determinants of cognitive health in this population. While the role of age resonates with global data, the influence of education offers an avenue for targeted interventions to delay or mitigate cognitive decline. Gender emerged as another factor warranting further investigation. Intriguingly, behavioral risk factors such as smoking and alcohol consumption did not show a significant association with cognitive impairment in our study, suggesting the need for further research to understand the specific sociocultural context. These findings have significant implications for healthcare planning, policy formulation, and intervention design, not only in Kerala but also in regions experiencing similar sociocultural transformations. The study calls for immediate attention to develop age- and education-specific healthcare strategies to improve the quality of life among elderly populations in old-age homes.
